# Supplementary figures and images for: Gene expression analysis suggests immunosuppressive roles of endolysosomes in glioblastoma
Source: PLoS One. 2024 Mar 20;19(3):e0299820. doi: 10.1371/journal.pone.0299820 (PMC10954093; doi:10.1371/journal.pone.0299820)

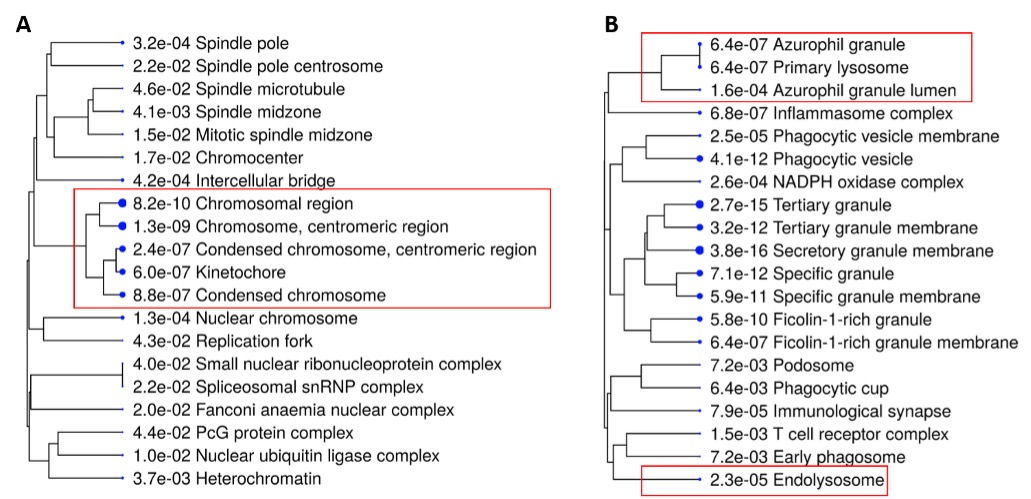

Supplement: S1 Fig — (A-B) The mRNA-seq data (protein-coding genes, tpm) of 162 TCGA GBM samples were subjected to quanTIseq analysis to estimate the fraction of each cell type. To identify genes that positively or negatively correlated with cancer cells, we calculated each gene’s correlation coefficient (r) between the gene expression level and the percentage of cancer cells in each GBM sample. The correlation coefficient cutoff for genes positively or negatively correlated with cancer cells was r > = 0.3 (n = 290) and r < = -0.5 (n = 347), respectively. (A) GO-Cellular Component enrichment analysis of genes positively correlated with cancer cells (annotated as “Other Cells”). (B) GO-Cellular Component enrichment analysis of genes negatively correlated with cancer cells (annotated as “Other Cells”). (A-B) Dendrograms show the top 20 enriched cellular components sorted by fold enrichment, and the size of each dot denotes the relative number of genes in each cellular component. (TIF) [file pone.0299820.s001.tif]

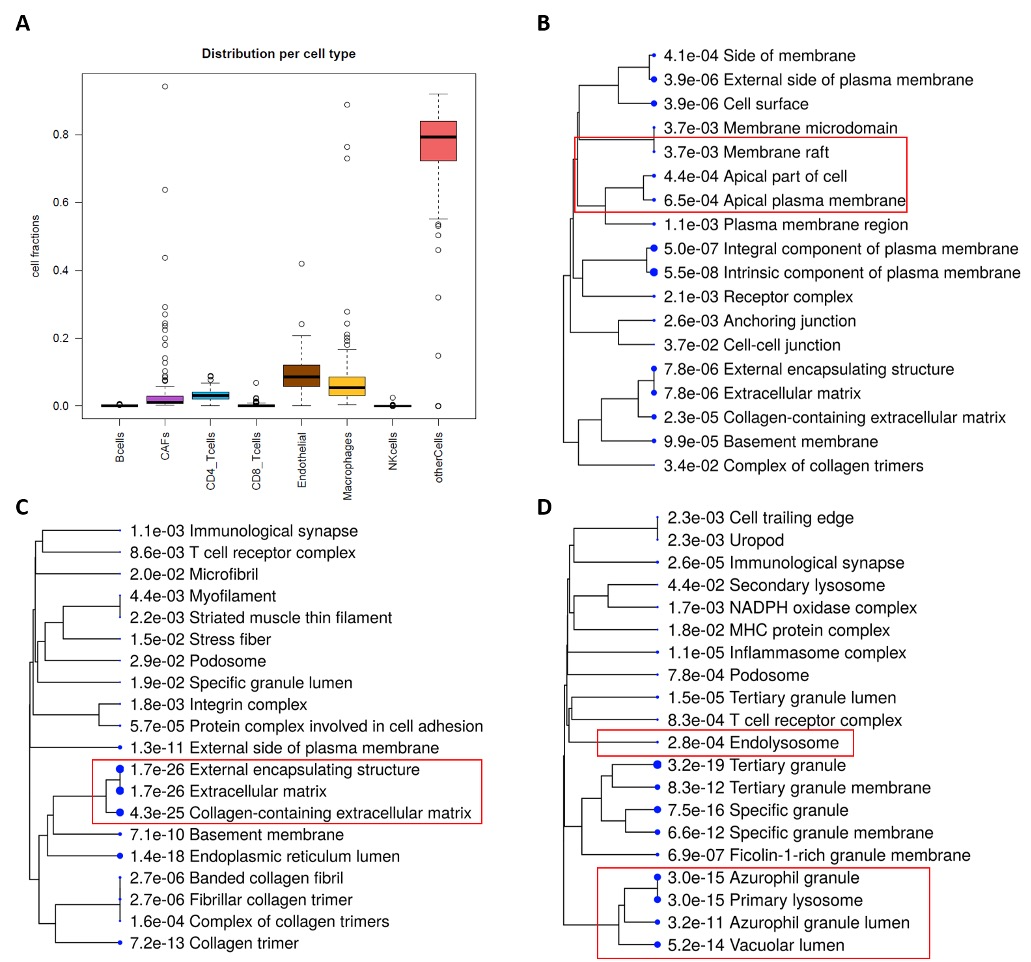

Supplement: S2 Fig — (A-C) The mRNA-seq data (protein-coding genes, tpm) of 162 TCGA GBM samples were subjected to EPIC analysis to estimate the fraction of each cell type. (A) Box plot showing the fractions of different cell types, including infiltrating immune cells and other cells (i.e., cancer cells), as estimated by EPIC. To identify genes that positively correlated with endothelial cells or CAF, we calculated each gene’s correlation coefficient (r) between the gene expression level and the percentage of endothelial cells or CAF in each GBM sample. The correlation coefficient cutoff for genes positively correlated with endothelial cells was r > = 0.3 (n = 237), and the cutoff for genes displaying strong positive correlation with CAF was r > = 0.5 (n = 543). (B) GO-Cellular Component enrichment analysis of genes positively correlated with endothelial cells. (C) GO-Cellular Component enrichment analysis of genes positively correlated with CAF. (D) The mRNA-seq data (protein-coding genes, tpm) of 162 TCGA GBM samples were subjected to xCELL analysis to estimate the fraction of each cell type, and genes positively correlated with macrophage (r > = 0.5) were used for GO-Cellular Component enrichment analysis. (B-D) Dendrograms show the top 20 enriched cellular components sorted by fold enrichment, and the size of each dot denotes the relative number of genes in each cellular component. (TIF) [file pone.0299820.s002.tif]

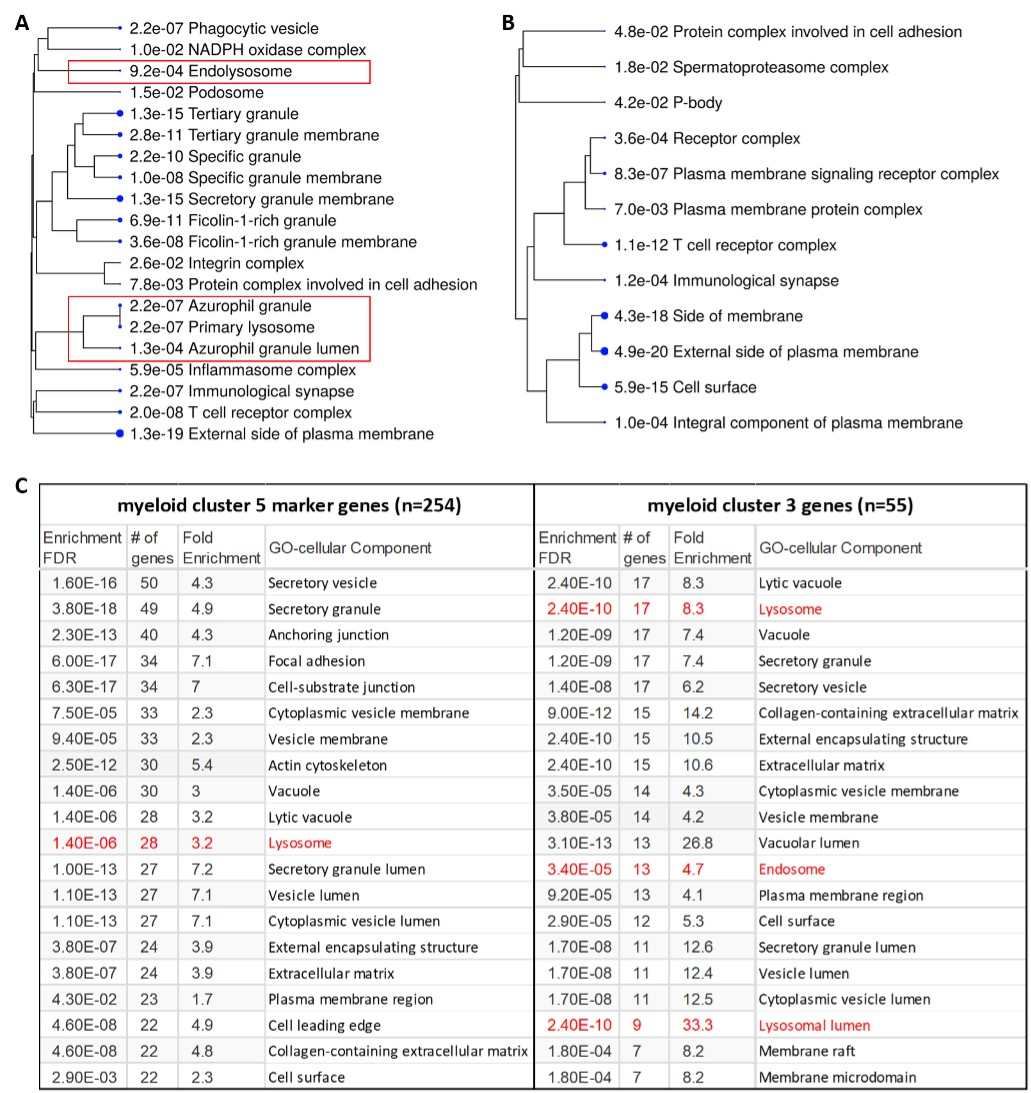

Supplement: S3 Fig — (A-B) The mRNA-seq data (protein-coding genes, tpm) of 162 TCGA GBM samples were subjected to CIBERSORT (absolute mode) analysis to estimate the fractions of M1 and M2 macrophages. To identify genes that positively correlated with M1 or M2 macrophages, we calculated each gene’s correlation coefficient (r) between the gene expression level and the percentage of M1 or M2 macrophages in each GBM sample. The correlation coefficient cutoff for genes positively correlated with M1 macrophages was r > = 0.3 (n = 318), and the cutoff for genes displaying strong positive correlation with M2 macrophages was r > = 0.5 (n = 429). Note that the cutoff value of r > = 0.3 was used for the M1 macrophage analysis to have the number of analyzed genes more comparable to the M2 macrophage analysis (when the cutoff value of r > = 0.5 was used, only 56 genes were identified). (A) GO-Cellular Component enrichment analysis of genes positively correlated with M2 macrophages. (B) GO-Cellular Component enrichment analysis of genes positively correlated with M1 macrophages. (A-B) Dendrograms show the significantly enriched cellular components (FDR cutoff = 0.05), and the size of each dot denotes the relative number of genes in each cellular component. (C) GO-Cellular Component enrichment analysis of marker genes for MC3 or MC5. The table shows the top 20 enriched cellular components sorted by the number of genes identified. Cellular components directly indicative of lysosomes/endosomes were highlighted in red. (TIF) [file pone.0299820.s003.tif]

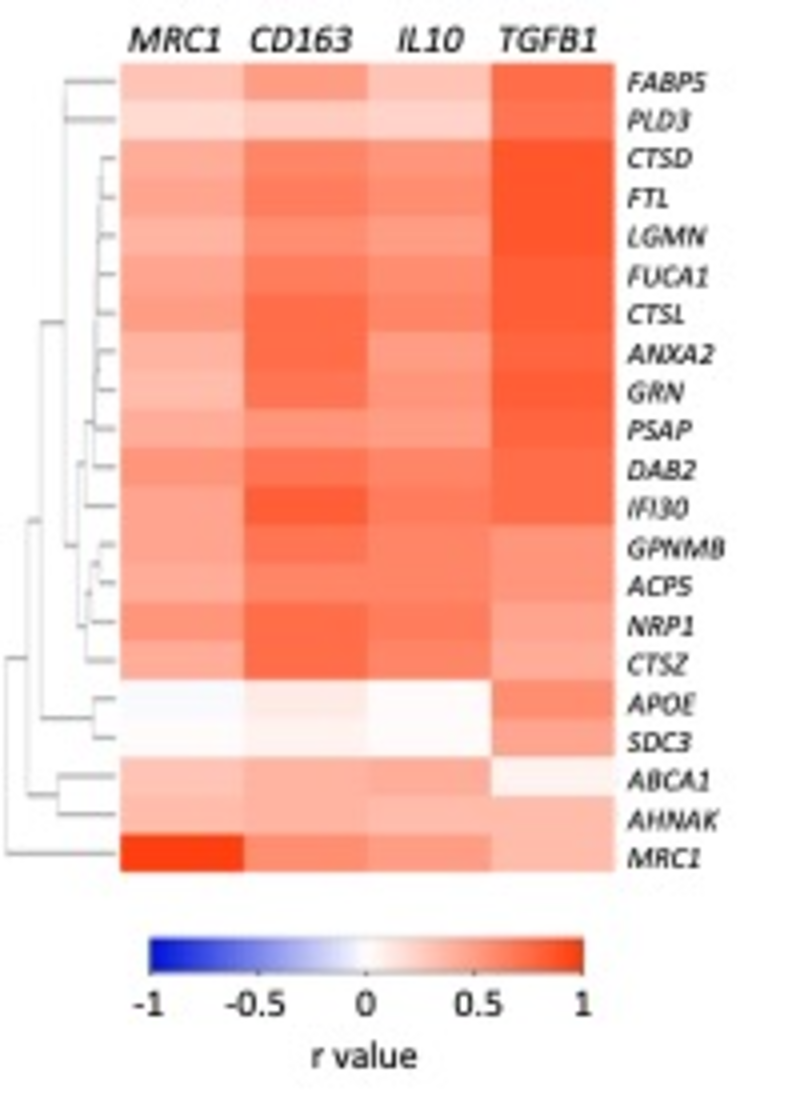

Supplement: S4 Fig — Heatmap demonstrating the Pearson correlation coefficient values (r-values) between the expression of 21 endolysosomal genes and immunosuppression markers MRC1 (CD206), CD163, IL10, and TGFB1. The heatmap was generated using Heatmapper (clustering method: single linkage; distance measurement method: Euclidean). The CGGA primary GBM dataset was used for the correlation analysis (n = 223). (TIF) [file pone.0299820.s004.tif]

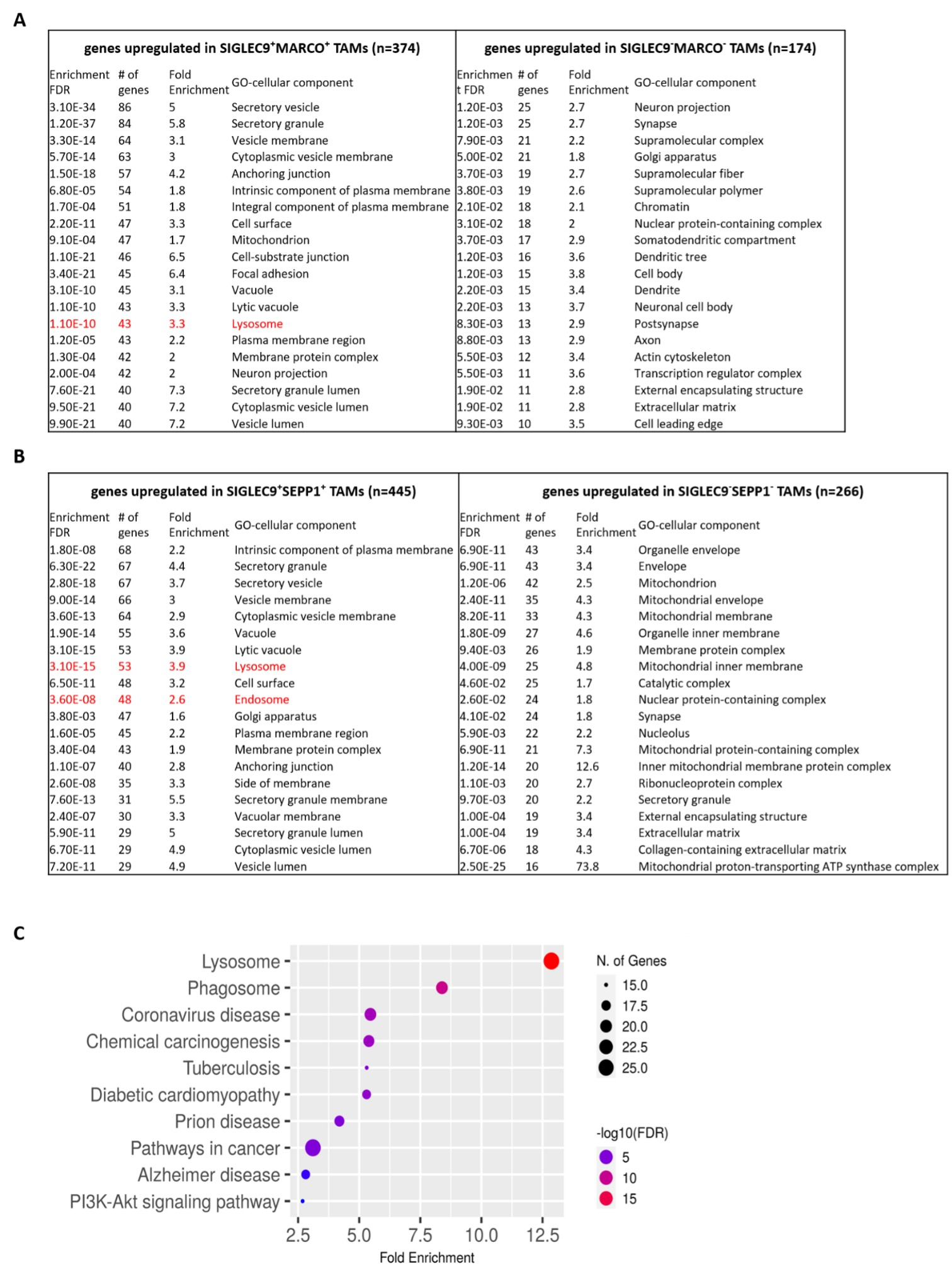

Supplement: S5 Fig — (A) GO-Cellular Component enrichment analysis of genes upregulated in SIGLEC9+MARCO+ TAMs (left panel) or SIGLEC9-MARCO- TAMs (right panel). (B) GO-Cellular Component enrichment analysis of genes upregulated in SIGLEC9+SEPP1+ TAMs (left panel) or SIGLEC9-SEPP1- TAMs (right panel). (A-B) Dendrograms show the top 20 cellular components sorted by the number of genes identified. Cellular components directly indicative of lysosomes/endosomes were highlighted in red. (C) KEGG pathway analysis of genes upregulated in SIGLEC9+SEPP1+ TAMs. The chart plot shows the top 10 enriched pathways (FDR cutoff = 0.05, sorted by fold enrichment). Similar analyses of genes upregulated in SIGLEC9+MARCO+ TAMs also identified lysosomes as significantly enriched. All known protein-coding genes were used as the background gene set. (A-C) For identifying upregulated genes, an FDR cutoff value of 0.05 was used for all analyses. (TIF) [file pone.0299820.s005.tif]
